# A Method for Assaying of Protein Kinase Activity In Vivo and Its Use in Studies of Signal Transduction in Strawberry Fruit Ripening

**DOI:** 10.3390/ijms221910495

**Published:** 2021-09-28

**Authors:** Wei Wang, Zhengrong Dai, Jie Li, Jinyao Ouyang, Tianyu Li, Baozhen Zeng, Li Kang, Kenan Jia, Zhiyuan Xi, Wensuo Jia

**Affiliations:** 1 College of Horticulture, China Agricultural University, Beijing 100193, China; w@cau.edu.cn (W.W.); BS20193170886@cau.edu.cn (Z.D.); S20193172448@cau.edu.cn (J.L.); S20203172650@cau.edu.cn (J.O.); litianyu@cau.edu.cn (T.L.); BS20203170857@cau.edu.cn (B.Z.); SY20203172717@cau.edu.cn (L.K.); SY20193172507@cau.edu.cn (Z.X.); 2 College of International Education, Beijing University of Chemical Technology, Beijing 100029, China; 18518635756@163.com

**Keywords:** strawberry, fruit ripening, protein kinase, FaMPK6, methyl jasmonate

## Abstract

Strawberry (*Fragaria × ananassa*) fruit ripening is regulated by a complex of cellular signal transduction networks, in which protein kinases are key components. Here, we report a relatively simple method for assaying protein kinase activity in vivo and specifically its application to study the kinase, FaMPK6, signaling in strawberry fruit. Green fluorescent protein (GFP)-tagged FaMPK6 was transiently expressed in strawberry fruit and after stimuli were applied to the fruit it was precipitated using an anti-GFP antibody. The precipitated kinase activity was measured in vitro using ^32^P-ATP and myelin basic protein (MBP) as substrates. We also report that FaMPK6 is not involved in the abscisic acid (ABA) signaling cascade, which is closely associated with FaMPK6 signaling in other plant species. However, methyl jasmonate (MeJA), low temperature, and high salt treatments were all found to activate FaMPK6. Transient manipulation of *FaMPK6* expression was observed to cause significant changes in the expression patterns of 2749 genes, of which 264 were associated with MeJA signaling. The data also suggest a role for FaMPK6 in modulating cell wall metabolism during fruit ripening. Taken together, the presented method is powerful and its use will contribute to a profound exploration to the signaling mechanism of strawberry fruit ripening.

## 1. Introduction

The strawberry (*Fragaria ananassa Duch.*) is an established experimental model for the study of fleshy fruit ripening, a complex process that involves substantial changes in numerous physiological and metabolic pathways. While many early studies of fruit ripening largely focused on the physiological and biochemical processes [1,2,3], more recent studies have targeted the underlying molecular mechanisms [4,5,6,7,8,9,10,11]. Such analyses have examined the roles of both structural and regulatory genes: the former encode proteins that directly control physiological and biochemical processes, and the latter act to control the behavior of the functional genes/proteins, and as such, make up complex signaling networks.

Reversible protein phosphorylation, catalyzed by protein kinases and phosphatases, is integral to a broad spectrum of cellular signal systems and mitogen-activated protein kinase cascades (MPKs), which are evolutionarily conserved signal transduction modules that are found in all eukaryotes. They play important roles in diverse aspects of plant growth and development, as well as hormone, biotic, and abiotic responses. Many studies have suggested that MPK cascades play key roles in signaling mediated by the hormone abscisic acid (ABA). ABA can regulate both the gene expression and protein activity of many other MPK pathway components. A number of studies have suggested that MPK cascades are involved in ABA-induced antioxidant defenses [12,13,14,15], in ABA-induced stomatal closure [16,17,18,19,20], and in ABA-induced suppression of seed germination [21,22,23,24,25,26]. The MPK family consists of serine/threonine protein kinases, and AtMPK6 from the model plant *Arabidopsis thaliana*, as well as homologs from other plant species, and have been implicated in diverse signaling cascades related to growth, development, and responses to abiotic and biotic stresses. We previously demonstrated that AtMPK6 is involved in ABA signaling [24,25,26] and there is evidence that ABI1, a key component of the ABA signaling pathway, physically interacts with AtMPK6, thereby inhibiting AtMPK6 activity [27] and providing direct evidence that AtMPK6 can serve as a key signal in ABA signal transduction.

There is considerable evidence that ABA also regulates strawberry fruit ripening [28,29,30,31,32,33], and we hypothesized that a homolog of AtMPK6 from strawberry (FaMPK6) might be involved in regulating fruit ripening. Traditional studies identifying gene function in fruit development and ripening have been largely based on the gain and loss of function strategies. However, to demonstrate whether a protein kinase may act as a key signal in hormone signal transduction, direct evidence would entail a demonstration of whether its activity would respond to ABA stimulation, and so the establishment of an in vivo protein kinase activity assay is a prerequisite.

The analysis of protein kinase activity, using in vivo or in vitro approaches, is a fundamental technique for studying phosphorylation-mediated signal transduction [34,35,36,37,38]. In vitro protein kinase assays can be used to characterize the phosphorylation by a protein kinase of its substrate, whereas in vivo protein kinase assays can reveal whether phosphorylation is controlled by particular signals or conditions. In-gel assays are common for in vivo protein kinase activity assays. However, there are several practical difficulties associated with using this method. Firstly, it is based on the denaturation and renaturation of the protein kinase, which can significantly deactivate the activity. Secondly, as the target protein kinase is not distinguished from other kinases, potential background noise may interfere with the analysis of the target kinase activity. Thirdly, this method requires both relatively large amounts of protein substrate and ^32^P-ATP, which is hazardous and requires careful handling in a lab setting. As regards to the in vivo assays of protein kinase in strawberry fruit, it would be much more difficult because of the difficulties in transgenic manipulation and protein extraction of tagged protein kinases in ripening fruit.

Here, we developed a method for the assaying of protein kinase activity in vivo based on its transient expression in strawberry fruit. The establishment of this method enables us to extensively explore fruit ripening-associated primary signals in relation to their regulation of FaMPK6 activity. Surprisingly, FaMPK6 was not found to be involved in ABA signaling, but rather was activated by jasmonic acid (JA), low temperature, and salt stress. This research not only sheds new light on the mechanisms of strawberry fruit ripening, but also adds to the compendium of techniques that can be used to unravel the mechanism of signal transduction in strawberry fruit.

## 2. Results

### 2.1. Optimization of Assays for Protein Kinase Activity In Vivo

**Figure 1:**
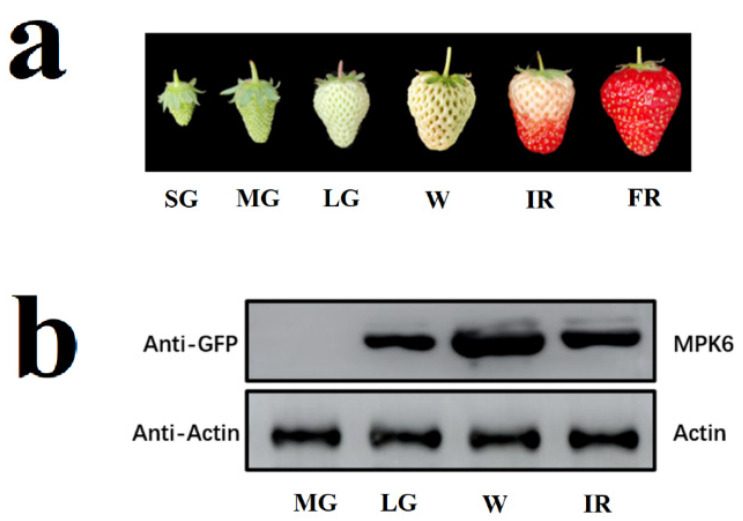
Accumulation of FaMPK6 following over-expression at different fruit developmental stages. (**a**) fruit phenotypes at different developmental stages. SG, small green; MG, middle green; LG, large green; W, white, IR, initially reddening, FR, fully reddening. (**b**) detached fruit at different developmental stages were transiently transformed with A. tumefaciens carrying a FaMPK6: eGFP fusion construct driven by the pSuper promoter. On day 4 after transformation, proteins were extracted from the fruit, and the amount of FaMPK6 was determined by immunoblotting with an anti-GFP antibody.

The assay of protein kinase activity in vivo depends on a large and quantitative accumulation of the target protein in tissues/organs. For transient gene expression to be used in the assay of protein kinase in strawberry, a major obstacle is its large variation among fruit samples. To deal with this problem, we first examined the potential factors that might interfere with protein accumulation driven by transient gene expression. FaMPK6 was fused to green fluorescent protein (GFP) that serves as a tag for both visualization and immunoprecipitation. Results showed that the MPK6 protein only accumulated after the LG (Large Green) fruit stage and that the maximum accumulation occurs at the W (White) fruit stage (Figure 1). Accordingly, fruit at the LG and W stage were used as the material in all the experiments presented here.

**Figure 2:**
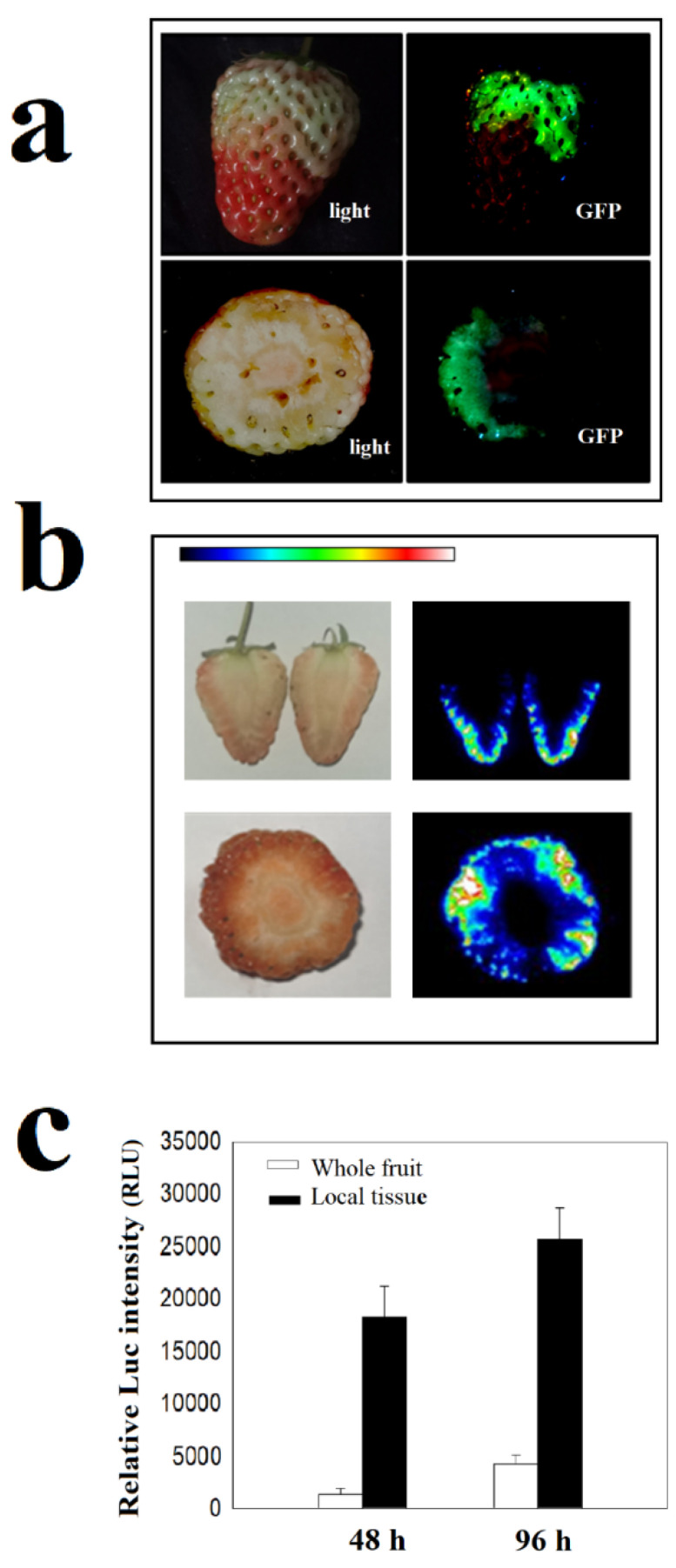
Distribution pattern of FaMPK6 within individual fruit. (**a**,**b**) detached fruit at the W stage were transformed with *A. tumefaciens* carrying either *FaMPK6: eGFP* (**a**) or *FaMPK6: Luc* (**b**) fusion constructs driven by the pSuper promoter. On day 4 after transformation, imaging was conducted. (**c**) detached fruit at the W stage were transiently transformed with *A. tumefaciens* carrying a *FaMPK6: Luc* fusion construct driven by the pSuper promoter. Luc activity was determined 48 h and 96 h after transformation. Whole fruit, protein was extracted from whole fruit; Local tissue, fruit tissue was excised from the areas that had been infiltrated with *A. tumefaciens*.

FaMPK6: GFP distribution was extremely uneven and was only observed in a very small area of the fruit. To quantitatively compare the amount of locally distributed protein to protein from the whole fruit, we examined the pattern of a FaMPK6 heterologously expressed as a fusion protein with luciferase (FaMPK6: Luc), as this fusion protein can be quantified based on Luc activity (Figure 2a,b). As shown in Figure 2c, the mean level of Luc activity in an entire fruit was five to ten times lower than in some localized areas, suggesting that in order to increase the concentration of the kinase protein, an effective strategy would be to use the specific tissues in the protein accumulated, in the image, rather than the whole fruit.

**Figure 3:**
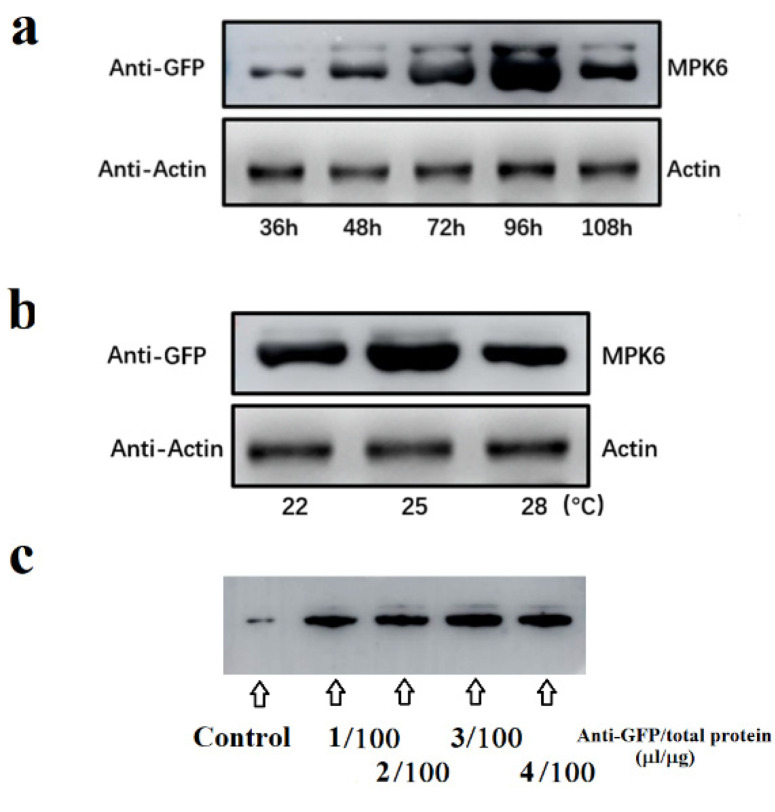
Optimization of FaMPK6 accumulation and immunoprecipitation. (**a**,**b**) FaMPK6 accumulation over a time course following *A. tumefaciens* infiltration and in response to different temperatures. Detached fruit at the W stage were transformed with *A. tumefaciens* carrying a *FaMPK6: eGFP* fusion construct driven by the pSuper promoter and FaMPK6 levels were assessed by immunoblotting. (**a**), immunoblot analysis showing the pattern of FaMPK6 over following transformation. (**b**) immunoblot analysis showing the pattern of FaMPK6 accumulation in response to different temperatures. After *A. tumefaciens* infection, detached fruit were incubated at different temperatures and on day 4 after incubation, FaMPK6 levels were determined. (**c**) pattern of FaMPK6 immunoprecipitation following incubation with different antibody concentrations. On day 4 after transformation, proteins were extracted and FaMPK6 was immunoprecipitated with different anti-GFP antibody concentrations. Precipitated proteins were examined by immunoblot analysis. Control, protein extract examined directly by immunoblotting without precipitation.

The initial accumulation was observed only 36 h after transformation, after which the level of recombinant protein greatly increased, although levels decreased after 100 h (Figure 3a). Maximal levels of the fusion protein were observed approximately 96 h after transformation. The highest levels were observed at 25 °C, although in comparison with developmental stage and transformation time, the effect of changing the temperature was much smaller (Figure 3b). Another consideration in the in vivo assay were factors affecting the immunoprecipitation. However, since this method is routine, if the quality of antibody is reliable, only antibody concentration needs to be considered. Figure 3c shows the pattern of the immunoprecipitation in relation to antibody concentration, and we observed that the titer exceeded a threshold, the amount of protein precipitated showed no further significant increase.

### 2.2. Screening for Internal and External Signals Upstream of FaMPK6

**Figure 4:**
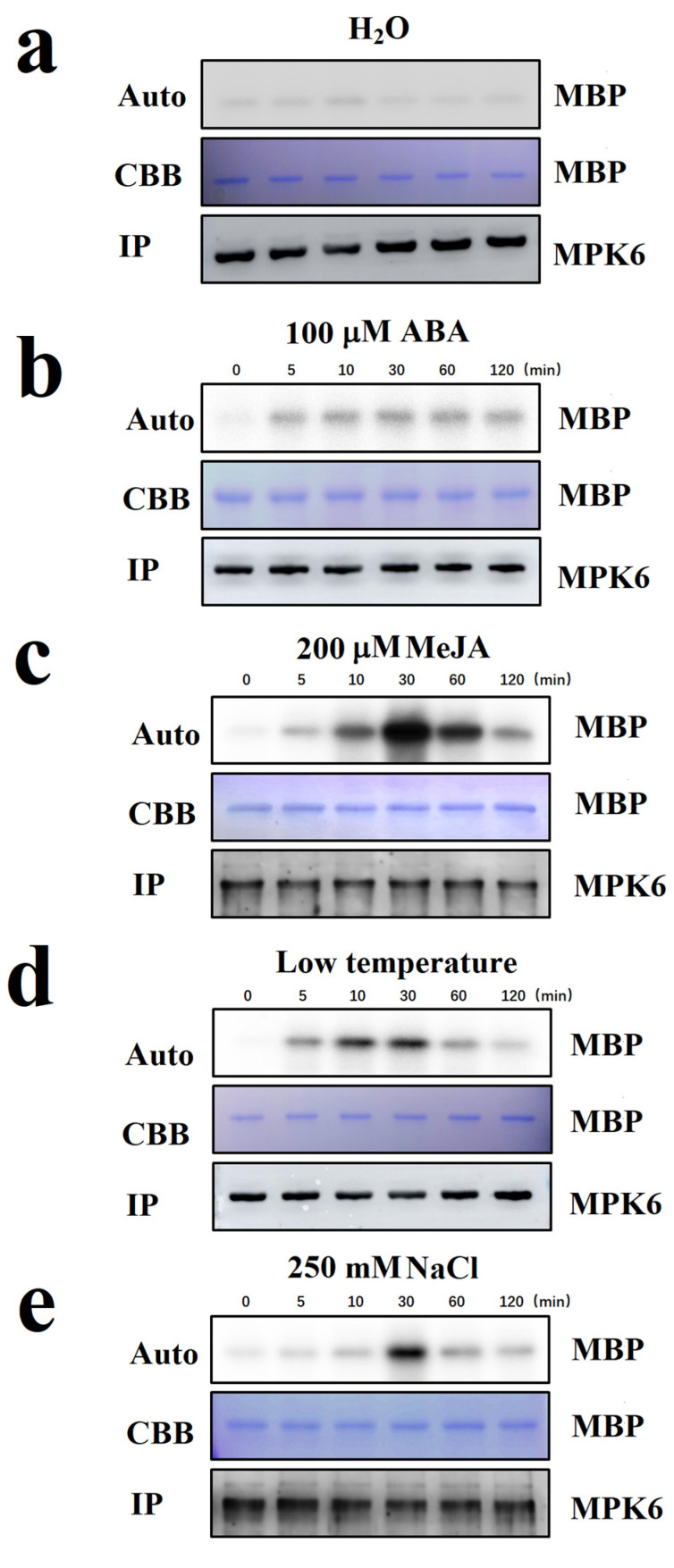
In vivo assaying of FaMPK6 activity in response to different stimuli. Detached fruit at the LG stage were transiently transformed with *A. tumefaciens* carrying a *FaMPK6: eGFP* fusion construct driven by the pSuper promoter. On the 4th day after transformation, fruits were treated, and then proteins were and the FaMPK6: GFP fusion protein was immunoprecipitated using an anti-GFP antibody. The in vitro kinase activity of FaMPK6 was measured using ^32^P-ATP and MBP as substrates. (**a**) control, water treatment; (**b**) 100 μM abscisic acid (ABA) treatment; (**c**) 200 μM methyl jasmonate (MeJA) treatment; (**d**) low temperature (4 °C); (**e**) high salt (250 mM NaCl) treatment.

FaMPK6 was not found to be responsive to the ABA treatment (Figure 4a,b). To determine whether this lack of response could be attributed to technical problem with the experimental procedure, and also to identify potential signals upstream of FaMPK6 signaling, we conducted a screen for signals that have previously been reported to be important for the regulation of strawberry fruit ripening. We observed that FaMPK6 activity increased within a few minutes in response to treatment with a functional analog of JA, methyl jasmonate (MeJA), and was substantially higher half an hour after the treatment (Figure 4c). We previously demonstrated that temperature is a key factor affecting strawberry fruit ripening [39] and so we also tested whether it might trigger FaMPK6 activity. This was indeed the case, and significant activation was observed only minutes after low temperate treatment (Figure 4d). In addition to its response to low temperature, MPK6 activity was also activated by salt stress, as demonstrated by incubating the fruit tissues in 250 mM NaCl solution for different times (Figure 4e). Taken together, these results suggest that FaMPK6 is involved in multiple fruit-ripening-associated signaling cascades.

### 2.3. FaMPK6 Functions in the Regulation of Phenylpropanoid-Associated Metabolism

**Figure 5:**
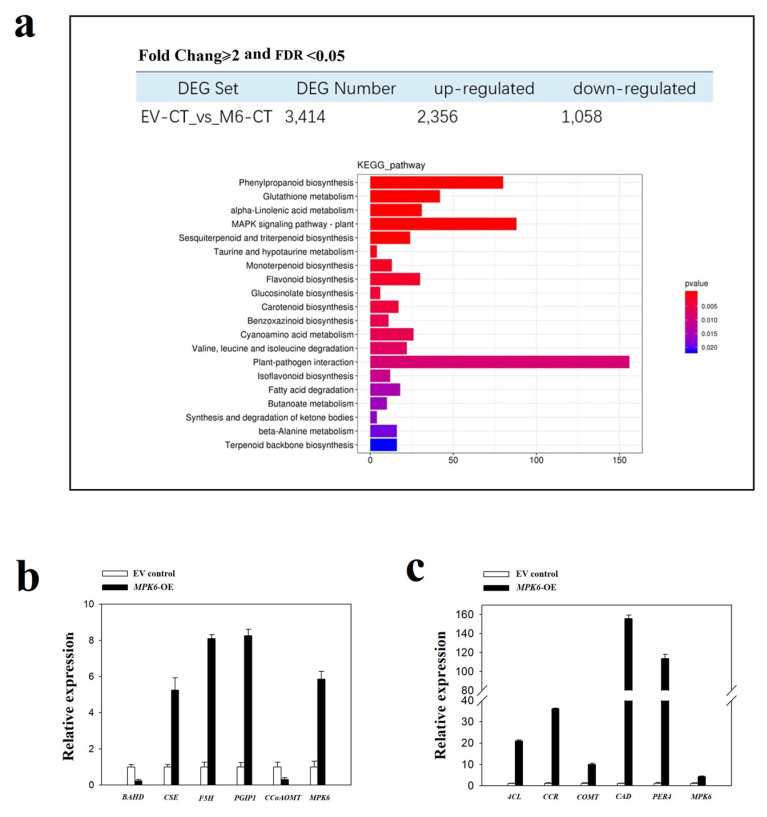
RNA-seq and quantitative real time (qRT)-PCR analysis. (**a**) expression pattern of genes affected by FaMPK6 over-expression. Detached fruit at the W stage were transiently transformed with *A. tumefaciens* carrying an empty vector or *FaMPK6* driven by the pSuper promoter. RNA-seq analysis was performed of extracts from fruit at day 4 after transformation. Genes regulated by *FaMPK6* were identified as having differences in transcript levels between the empty vector control (EV) and *FaMPK6* over-expressing fruits at a fold change ≥ 2 and a false discovery rate (FDR) < 0.05. RNA-seq analysis was performed of pools of 10 individual fruit, with three biological replicates. (**b**,**c**) qRT-PCR analysis (a pool of 10 individual fruit with three biological replicate) showing the expression pattern of several marker genes implicated in phenylpropanoid metabolism as affected by *FaMPK6* over-expression. (**b**) genes with fold changes < 10; (**c**) genes with fold changes > 10. Error bars are mean ± SD of three biological replicates.

RNA-seq analysis of strawberry fruit over-expressing *FaMPK6* caused an increase/decrease in the transcription of 2356/1058 genes, and the *Kyoto Encyclopedia of Genes and Genomes* (KEGG) analysis of the annotations of the genes identified approximately 20 metabolic pathways that are affected by *FaMPK6* over-expression (Figure 5a). These included pathways associated with phenylpropanoid biosynthesis, glutathione metabolism, linolenic acid metabolism, as well as plant-pathogen interactions. A further search of the transcriptome data identified > 80 genes implicated in phenylpropanoid metabolism (Supplemental Figure S1). Figure 5b,c show the expression pattern of genes from some of these families and the effect of *FaMPK6* over-expression. While BAHD acyltransferase and Caffeoyl coenzyme A dependent O-methyltransferase (*CCoAOMT*) expression was lower in the transgenic fruit than in wild type (untransformed) fruit, the expression of Caffeoylshikimate esterase (*CSE*), Ferulate 5-hydroxylase (*F5H*), Polygalacturonase-inhibiting protein (*PGIP*), 4-coumarate-coenzyme A ligase (*4CL*), Cinnamoyl-CoA reductase (*CCR*), Caffeate O-methyltransferase (*COMT*), Cinnamyl alcohol dehydrogenase (*CAD*) and Lignin-forming anionic peroxidase 4 (*PER4*) genes increased in response to *FaMPK6* over-expression.

### 2.4. FaMPK6 Functions in the Regulation of Fruit-Ripening Associated Metabolism

**Figure 6:**
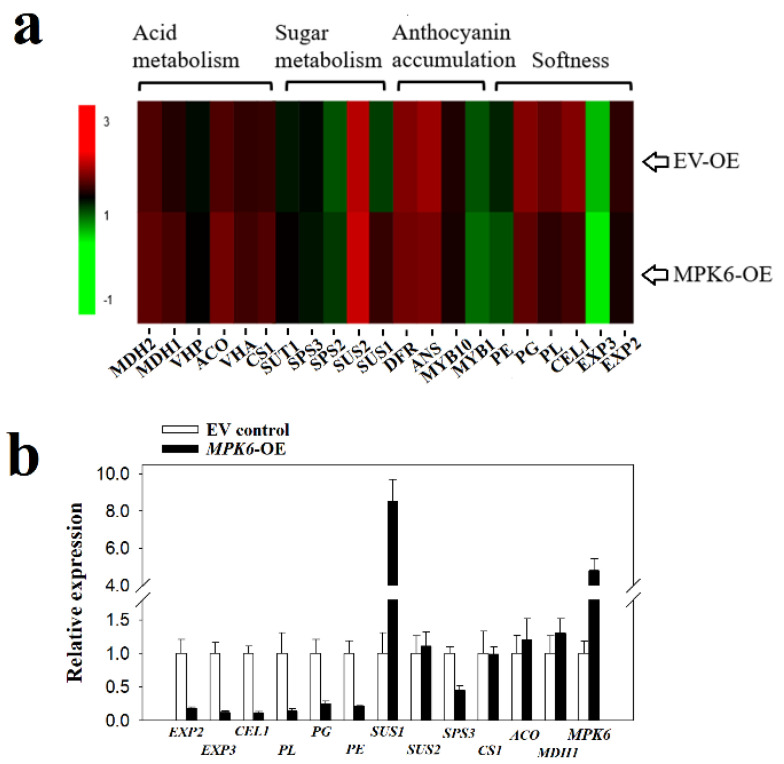
Expression pattern of ripening-associated maker genes as affected by FaMPK6 over-expression. (**a**) heat map showing the expression pattern of ripening-associated maker genes affected by *FaMPK6* over-expression. Detached fruit at the LG stage were transiently transformed with *A. tumefaciens* carrying empty vector or *FaMPK6* driven by the pSuper promoter. On day 4 after transformation, RNA-seq analysis was performed of a pool of 10 individual fruit, with three biological replicates. (**b**) quantitative real time qRT-PCR verification of the RNA-seq data. qRT-PCR analysis was also performed on a pool of 10 individual fruit with three biological replicates. Error bars indicate the mean ± SD of three biological replicates.

Fruit ripening is a complex process involving dramatic major changes in many biochemical pathways, including those involved in sugar, organic acid, anthocyanin metabolism, and cell wall modification. The expression of some genes associated with these processes, such as expansin genes (*EXP2*, *EXP3*) and pectate lyase (*PL*), and a polygalacturonase (*PG*), decreased as a consequence of *FaMPK6* over-expression, whereas no significant difference was observed for many other genes, such as Sucrose synthase 2(*SUS2*), Citrate synthase 1(*CS1*), Aconitate hydratase (*ACO*), and Malate dehydrogenase 1 (*MDH1*), which suggests that FaMPK6 might be a signal primarily regulating cell wall metabolism, rather than a signal controlling overall fruit ripening (Figure 6).

### 2.5. FaMPK6 Functions in the Mediation of JA-Induced Gene Expression

Given the high sensitivity of *FaMPK6* expression to MeJA treatment, we further investigated the role of *FaMPK6* in the mediation of JA-induced gene expression. We examined the genes that were affected by MPK6 over-expression and RNAi down-regulation.

**Figure 7:**
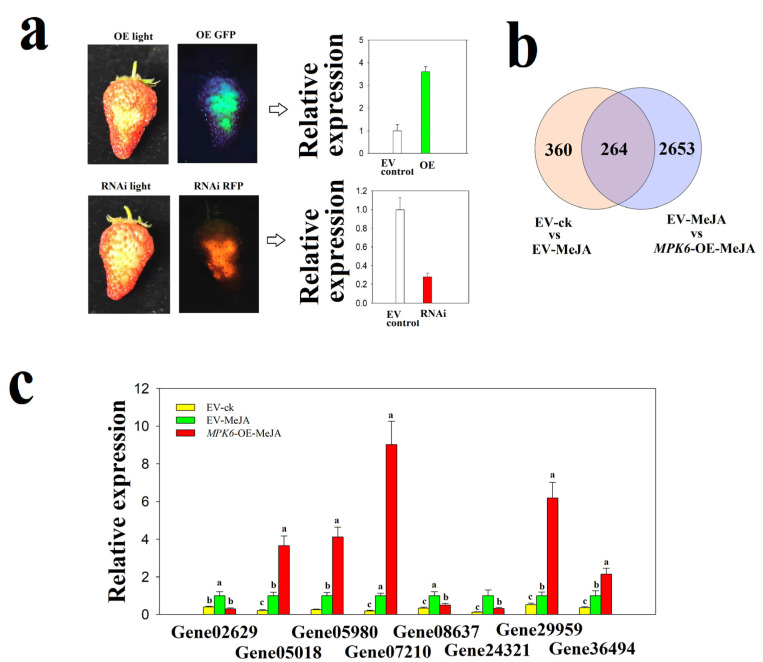
Expression pattern of genes regulated by *FaMPK6* in the jasmonic acid (JA) signaling pathway. (**a**) the upper left image shows the pattern of *FaMPK6* over-expression, as indicated by green fluorescent protein (GFP) image analysis of a FaMPK6: GFP fusion protein and qRT-PCR analysis of the *FaMPK6* expression pattern in these fruit; the lower left image shows *FaMPK6* RNAi down-regulation, as indicated by red fluorescent protein (RFP) image analysis of a *FaMPK6* RNAi construct carrying the *RFP* gene driven by 35S promoter and qRT-PCR analysis of the *FaMPK6* expression pattern in these fruit. (**b**) Venn diagram showing the expression pattern of genes regulated by methyl jasmonate (MeJA) and *FaMPK6.* (**c**) qRT-PCR verification of several genes controlled by *FaMPK6* in the JA signaling pathway. Detached fruit at the LG stage were transiently transformed with *A. tumefaciens* carrying an empty vector, *FaMPK6*-OE or *RNAi* constructs. On day 4 after transformation, RNA-seq analysis, imaging and qRT-PCR analysis were performed. Error bars represent the mean ± SD of three biological replicates. Significant difference in different treatment for each gene is indicated by different letters (*p* < 0.05).

FaMPK6 transcript levels were higher or lower, respectively, in the over-expressing and RNAi down-regulated fruit, compared with the empty vector control (EV control) fruit (Figure 7a). RNA-seq analysis indicated that *FaMPK6* over-expression caused significant changes in the expression pattern of 2749 genes, of which 264 genes are associated with MeJA signaling based on their annotations (Figure 7b; Supplemental Figure S2). Of these 264 genes, the expression of some increased while others decreased following treatment with MeJA, indicating that FaMPK6 acted as both a positive and a negative signal in the JA signaling pathway). To validate the pattern of gene expression of RNA-Seq analysis, several representative genes that show dramatic changes in response to MeJA treatment or FaMPK6 over-expression were further examined for their expression by qRT-PCR, which shows similar patterns as indicated by the RNA-Seq analysis (Figure 7c).

## 3. Discussion

### 3.1. Importance of In Vivo Protein Kinase Activity Assay Method Development

In recent years, studies of the molecular mechanisms involved in fruit ripening have focus on the structural genes that function in the regulation of physiological and biochemical metabolic pathways [4,5,6,7,8,9,10,11], and there is relatively little information regarding the underlying signaling mechanisms. One obstacle to the elucidation of fruit ripening-associated signal transduction has been the absence of appropriate in vivo protein kinase activity assays. The terminology ‘in vivo’ means that a protein kinase activity can be evaluated for its cellular response in response to signal stimuli, which is in contrast to the method by which a protein kinase expressed in vitro are determined for its activity towards substrates. In this study, with FaMPK6 as an example, we have developed a method by which protein kinase activities can be determined for their cellular responses to signal stimuli. In this method, GFP-fused *FaMPK6* was expressed in strawberry fruit, and after signal stimulus, it was isolated by immuno-precipitation and its activity was determined in vitro. Although the FaMPK6 activity was determined in vitro in the last step, it has no essential difference from the commonly used In-gel kinase assay [40,41], because both methods mean to evaluate protein kinase activity in response to signal stimuli. The current protocol for assaying of protein kinase activity in vivo is based on the transient expression of the gene encoding the tagged protein kinase and protein immunoprecipitation in/from strawberry fruit. Compared to the in-gel method, current protocol is much easier and sensitive, because it has avoided the protein denaturation and renaturation in the In-gel assay.

### 3.2. Broader Use of the In Vivo Protein Kinase Activity Assay

The method development in this study was based on the determination of FaMPK6 activity in *Fragaria × ananassa* Duch, Benihoppe. An important question is whether it is applicable to other protein kinases or other strawberry varieties. The method is essentially based on two key techniques: transient gene expression in the fruit, and immunoprecipitation of proteins from the transformed fruit. Agro-infiltration is a well-established technology which has been successfully applied in several different projects as a rapid assay for gene function analysis [42,43]. Nevertheless, this does not imply that it can be easily used for assays of protein kinase activity in vivo, because kinase assay in vivo depends on a large and quantitative accumulation of the target kinase, but the transient expression in strawberry fruit is quite variable due to a number of factors affected [33]. Accordingly, to develop the kinase assay method, we first evaluated some factors that may possibly affect the accumulation of the kinase protein. We observed that the large variation can be resolved by determining a suitable sampling strategy [39]. Regarding strawberry varieties, as transient gene expression has already been described in several different strawberry varieties [33], the current protocol should be applicable to different strawberry varieties, although this was not specifically investigated in the current study.

As mentioned above, the current protocol is much easier than the commonly used in-gel kinase assay method. This enables the possibility to extensively screen the protein kinases involved in the regulation of strawberry fruit ripening. Notably, aside from analyzing protein kinases for their activity responses to signal stimuli, the current method can be also used for unravelling signaling pathways. This can be achieved by expressing a tag-fused protein kinase in strawberry fruit with upstream candidate signal knocked out by stably genetic transformation. As such, this research would contribute greatly to a profound exploration into the signaling mechanisms for strawberry fruit ripening.

Assays of protein kinase activity often involve measuring ^32^P transferred from ^32^P-ATP to the protein substrate. In such cases, accurate measurements of protein kinase activity largely depend on the effective precipitation of the kinase. In the current study, to precipitate the target kinase we adopted a strategy in which FaMPK6 was fused to a GFP tag, and demonstrated that the fusion protein was precipitated while the activity remained high (Figure 4). Other peptide tags, such as FLAG and MYC, might also be used.

### 3.3. Key Points for In Vivo Assaying of Protein Kinase Activity

An important factor in the success of the method described here is the optimization of transient gene expression. We previously examined the experimental conditions that may have a major effect on transient gene expression in the fruit [33]. As the gene expression patterns were not definitely identical to those of protein accumulation due to protein degradation, we examined several variables that may potentially affect protein accumulation (Figure 1, Figure 2 and Figure 3). Similar to the pattern of gene expression, the protein accumulation is also mainly limited by the fruit developmental stages. Protein was accumulated only after the large green stage. Besides the limitation of developmental stage, to measure kinase activity, sufficient amounts of protein must be precipitated and since recombinant protein accumulation is extremely uneven within individual fruit (Figure 2), we propose using only tissues with strong protein accumulation. These can be identified by isolating tissue blocks that show high levels of a reporter signal, such as GFP.

Another key point is a suitable sampling strategy. Our assay is aimed at demonstrating whether a kinase activity can respond to different stimuli, which is in turn based on extracting equivalent amounts of protein from different samples. However, we observed great variation in recombinant protein accumulation. This problem can be addressed by using a pooled sample, consisting of tissue from many fruits. According to our recent study, to achieve an acceptable error margin, a pool of at least five to 10 fruits, with at least three biological replicates of each, is required [39]. In the present study, we used 10 fruit in each pooled sample and showed that the FaMPK6 concentration was similar between different samples (Figure 3 and Figure 4). The immunoprecipitation step is widely used and does not require much optimization. However, the antibody titer may be an important consideration. As shown in Figure 3c, we saw little difference in the precipitation efficiency when the antibody concentration exceeded a certain threshold, and the use of a relatively low concentration might be both more cost effective and reduce background noise.

### 3.4. FaMPK6 Contributes to the Regulation of Secondary Wall Metabolism during Fruit Ripening

While the current study aims to develop a method for measuring protein kinase activity in vivo, whether the so-developed method can be practically used for the identification and characterization of the protein kinases involved in the regulation of fruit ripening needs to be confirmed. JA has been established to play a critical role in the regulation of strawberry fruit ripening [44]. Given that FaMPK6 was observed to be capable of sensitively responding to JA stimulus, to demonstrate the applicability of the current method, we further investigated whether FaMPK6 indeed played a role in the regulation of strawberry fruit ripening. Comparative RNA-seq analysis of fruit overexpressing FaMPK6 and control fruit suggested that FaMPK6 contributes to the regulation of secondary wall metabolism (Figure 5). Recently, we demonstrated that strawberry fruit ripening is initiated by primary wall degradation [45].Moreover, secondary wall degradation occurs following disassembly of the primary wall [45], which suggests that FaMPK6 functions in the regulation of late fruit ripening events.

ABA has been implicated as a key signal controlling ripening in several fruit, including strawberry [33,34,35,36,37,38]. Since MPK6 has been demonstrated to physically interact with the ABA coreceptor ABI1, we hypothesized that *FaMPK6* regulates strawberry fruit ripening via ABA signaling. However, in vivo protein kinas activity assays indicated that FaMPK6 is not involved in ABA signaling, but rather contributes to JA signaling, implying that FaMPK6 acts to regulate strawberry fruit ripening via JA signaling. These observations shed new light on the regulatory mechanisms involved in strawberry fruit ripening and highlight the potential of the in vivo kinase assay as platform for biological discovery.

### 3.5. FaMPK6 Mediates Multiple Signaling Cascades

We found that FaMPK6 activation was highly sensitive to many internal and external cues, such as temperature, salt stress and JA, all of which have also been implicated in strawberry fruit ripening regulation (Figure 4). Given its effect on many fruit ripening associated genes, we conclude that FaMPK6 mediates multiple signaling cascades involved in fruit ripening regulation. However, we did not observe any significant difference in fruit coloration in either FaMPK6 over-expressing or RNAi silenced fruit compared with control fruit. This might reflect the limitations of the transient gene expression, since it takes a relatively long time for genes to be expressed at high levels, and conversely a relatively short time for the fruit to change color. However, these observations indicate that FaMPK6 acts to partially control fruit ripening rather than to control the initiation of fruit ripening. As shown in Figure 4, while FaMPK6 was activated by multiple stimuli, and it will be interesting elucidate the uncharacterized JA-FaMPK6 signaling cascade.

## 4. Materials and Methods

**Plant materials:**

Octoploid strawberry plants (*Fragaria × ananassa* Duch., Benihoppe) were grown in a greenhouse at 18–28 °C and 75–90% humidity under an 8-h dark/16-h light cycle. The strawberry fruit were classified into six developmental stages: SG, small green; MG, middle green; LG, large green; W, white, IR, initially reddening, FR, fully reddening.

**Reagent and buffers:**

Agar, tryptone, and yeast extract were purchased from OXOID (Basingstoke, Hants, UK). PMSF, EDTA, DTT, Triton X-100, and glycerin were purchased from Thermo Scientific (USA), Protease Inhibitor Cocktail (1×) were purchased from Cwbio Biotech Company (Beijing, China), and MeJA, ABA, and acetosyringone were purchased from Sigma (St.Louts, MO, USA). Antibiotics, including Ampicillin, Kanamycin, Spectinomycin, and Rifampicin, were purchased from Sigma (St.Louts, MO, USA). TransStart^®^ FastPfu PCR SuperMix (high-fidelity enzyme) was purchased from TransGen Biotech (Beijing, China). Restriction endonuclease and T4 DNA ligase were purchased from NEB (Ipswich, MA, USA). DH5α chemically competent cells and *Agrobacterium* GV3101 competent cells were purchased from TransGen Biotech Company (Beijing, China) and Biomed Biotech Company (Beijing, China), respectively.

**Fruit transient gene expression:**

Fruit transient gene expression was performed as previously described [33] with minor revisions. For gene functional analysis, approximately 0.5 mL of the *Agrobacterium tumefaciens* culture carrying either empty vector or target vector were individually injected into one half of an intact fruit with only one injection per fruit. On day four after *A. tumefaciens* transformation, fruit were cut in half. Five halves injected with empty vector were collected as one control sample, and five halves injected with target vector were collected as one gene manipulation sample. To generate material for immunoblot blot analysis and in vivo assays of protein kinase activity, stages of fruit were injected with the *A. tumefaciens* carrying the target vector. Samples were collected at various times after transformation or after the various treatments (five fruit were mixed as an individual sample).

**RNA extraction and quantitative real time (qRT)–PCR:**

RNA extraction and qRT–PCR were performed as previously described [33]. Briefly, total RNA was extracted from fruit using the Plant RNA kit (Omega, R6827-01, Norcross, GA, USA). RT-PCR was conducted according to the NovoScript^®^ Plus All-in-one 1st Strand cDNA Synthesis SuperMix protocol (gDNA Purge) (Novoprotein, E047-01A, Shanghai, China). qRT–PCR was performed on a QuantStudio 6 Flex Real-time PCR system using Biomarker 2X SYBR Green Fast qPCR Mix (Biomarker, RK02001, Beijing, China). The primers are listed in Supplemental Table S1.

**Vector construction:**

The pSuper-1300-GFP vector, carrying the GFP reporter driven by the Super promoter, was used to construct MPK6-GFP. The pSuper-1300-Fluc vector, which carries the Fluc reporter driven by the Super promoter, was used to construct MPK6-Fluc. The pK7GWIWG2 II-RedRoot vector was used to construct MPK6-RNAi.

The full-length coding sequence of FaMPK6 was amplified from *Fragaria* × *ananassa* Duch. (Benihoppe) cDNA using the MPK6-BS-F (5′-GGATCCATGGAAGTCGGAGGTCAATCAGG-3′) and MPK6-BS-R (5′-GTCGACATGCTGCTGATACTCAGGGTTAA-3′) primers, and the PCR product was cloned into the *Bam*HI and *Sal*I sites of the pSuper-1300 vector to obtain the MPK6-GFP and MPK6-Fluc vectors. To produce the MPK6-RNAi vector, a MPK6 fragment (nucleotides 126 to 371) was amplified using the MPK6-RNAi-F (5′-AAAAAGCAGGCTT CCAGTACAACATCTTCGGCAAC-3′) and MPK6-RNAi-R (5′-AGAAAGCTGGGTA GATAATGTCCCGAATGGCAAC-3′) primers. The PCR product was introduced into the pK7GWIWG2 II-RedRoot vector using Gateway technology [46].

**Immunoblot analysis:**

Immunoblot analysis was performed as previously described [33] with minor revisions. Briefly, fruit proteins were extracted with a buffer containing 50 mM Tris-HCI (pH7.4), 250 mM NaC1, 0.1% (*v*/*v*) Triton X-100, 2 mM EDTA, 10% glycerol and Protease Inhibitor Cocktail (1×). Protein samples were separated by SDS-PAGE electrophoresis and immunoblot analysis was performed using an anti-GFP monoclonal antibody (ABclonal, AE012).

**Imaging and quantitative analysis of GFP and LUC:**

Imaging of GFP fluorescence was performed using instrumentation built in-house that has an argon laser, a 488-nm excitation filter, and a 507-nm emission filter, which allows up to 100 cm^2^ to be imaged at a time. For LUC imaging, fruit were sprayed evenly with luciferase reaction solution, and image analysis was performed using a LUCK fluorescence analyzer. LUC activity was determined according to the protocol described by Zeng et al. [44] with minor modification. Briefly, luciferase reaction reagent was equilibrated to room temperature and thoroughly mixed with protein extract. The reaction mixture was transferred into an opaque 96 well plate and the activity was measured by a luminometer (FL × 800, BioTek, Winooski, VT, USA) and expressed as Relative Light Unit (RLU).

**RNA-Seq:**

RNA-seq was utilized to investigate the gene-expression patterns in EV-OE and MPK6-OE fruits. RNA integrity was assessed using the RNA Nano 6000 Assay Kit of the Agilent Bioanalyzer 2100 system (Agilent Technologies, Santa Clara, CA, USA). A total of 1 μg RNA was used per sample as input material for the RNA sample preparations. Sequencing libraries were generated using NEBNext UltraTM RNA Library Prep Kit for Illumina (NEB) following manufacturer’s recommendations and index codes were added to each sample. After cluster generation, the libraries were sequenced on an Illumina platform and paired-end reads were generated. Three independent experiments were performed to collect RNA-seq data. Approximately 6.0 GB of clean-read data were generated from each sample. Hisat2 tools soft were used to map with reference genome. The DEG (differentially expressed genes) data were determined using DESeq [47] to identify genes showing a fold change ≥ 2 and a false discovery rate (FDR) ≤ 0.05. We used the KOBAS [48] software to test the statistical enrichment of DEGs from KEGG pathways [49].

**Hormone and abiotic stress stimuli:**

To examine FaMPK6 activity in response to hormone and abiotic stress stimuli, detached fruits were transformed with *A. tumefaciencs* carrying *FaMPK6* fused with *GFP* tag or the empty vector (control). Four days after the transformation, the fruit tissues were treated with 200 μM MeJA, 200 μM ABA or 250 mM NaCl. To balance the biotic background among the tissues for different stimuli, individual fruit was evenly divided into four parts and the four parts coming from 10 individual fruits were mixed as one individual sample. Individual samples were then infiltrated with 200 μM MeJA using a vacuum pump, and then further incubated for different times at ambient temperature. Samples were frozen in liquid nitrogen and stored at −80 °C until use.

To examine the pattern of MeJA induced-gene expression as affected by *MPK6* over expression, half part of individual fruit was transformed with *A. tumefaciencs* carrying either *FaMPK6* fused with *GFP* (over expression, OE) or empty vector (control, EV). Four days after transformation, the fruits were cut into halves and treated with 200 μM MeJA as described above.

**Protocol for in vivo assays of protein kinase activity:**

Step 1. Transient gene expression and protein extraction

We first constructed a vector carrying the sequences for the FaMPK6 target kinase in-frame with the GFP tag sequence at C-terminus. We have found that GFP is an idea tag that is well immunoprecipitated by a specific antibody. Tissue blocks showing fluorescence are cut out from 10 fruits to generate a pool constituting one sample. Proteins were extracted from the fruit samples by first powdering the samples in liquid nitrogen and then adding lysis buffer (50 mM Tris-HCI (pH 7.4), 250 mM NaC1, 0.1% (*v*/*v*) Triton X-100, 2 mM EDTA(PH8.0), 10% glycerol, 5 mM NaF, and 1 mM PMSF, Protease Inhibitor Cocktail (1×)) in a ratio of 1:3 (*v*/*v*) and incubating at 4 °C for 30 min. Samples were then centrifuged at 12,000× *g* at 4 °C for 10 min and the supernatants collected and pooled.

Step 2. Immunoprecipitation

Proteins were immunoprecipitated by adding different amounts of Anti-GFP mAb-Agarose (MBL, D153-8) to give different titers. Normally, a ratio of 1 mg protein/10 mL antibody can be used. The sample was mixed well and incubated with gentle agitation for at least 60 min at 4 °C. The beads were washed twice with buffer [20 mM Tris-HCl (pH7.4), 2 mM EDTA, 100 mM NaCl, and 0.1% Triton X-100], and centrifuged at 2000× *g* for 1 min. The beads were washed with the PBS (pH 7.4), centrifuged at 2000× *g* for 1 min and finally washed with kinase buffer [20 mM HEPES (pH 7.4), 10 mM MgCl_2_, 5 mM EGTA, and 1 mM DTT].

Step 3. In vitro phosphorylation assay

The beads were re-suspended in 30 µL kinase buffer containing 2 μg myelin basic protein (MBP) (Sigma, M1891), 25 μM ATP, and 1 μCi [γ-^32^P]-ATP. The protein kinase reactions were performed at 25–30 °C for 30 min and the reactions were stopped by addition of 5 × SDS loading buffer, and boiling for 5–10 min. The reactions were then centrifuged at 12,000× *g* for 10 min and the supernatants fractionated by SDS-PAGE. The resulting gels were imaged using a Typhoon FLA 9500 phosphor imager (Amersham, Boston, MA, USA).

## 5. Conclusions

Strawberry is an established experimental model for the study of fleshy fruit ripening. However, a lack of method for assaying of protein kinase activity in vivo has been limiting the investigation of the signaling mechanisms controlling strawberry fruit ripening. This study has established a powerful method for assaying of protein kinase activity in vivo, which will contribute greatly to a profound unraveling of the mechanisms behind the regulation of fleshy fruit ripening.

## Supplementary Materials

The following are available online at https://www.mdpi.com/article/10.3390/ijms221910495/s1.
