# Supplementary material for: A Method for Assaying of Protein Kinase Activity In Vivo and Its Use in Studies of Signal Transduction in Strawberry Fruit Ripening
Source: Int J Mol Sci. 2021 Sep 28;22(19):10495. doi: 10.3390/ijms221910495 (PMC8508642; doi:10.3390/ijms221910495)
Supplement: Supplementary file 1 [file ijms-22-10495-s001.zip › ijms-1367522-supplementary.pdf]

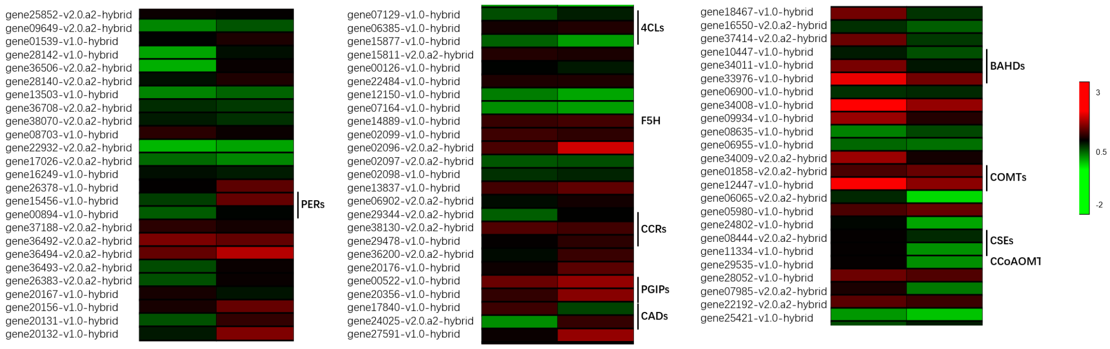

Supplementary Figure S1. heatmap showing expression levels of genes implicated in phenylpropanoid metabolism that were affected by FaMPK6 over-expression.

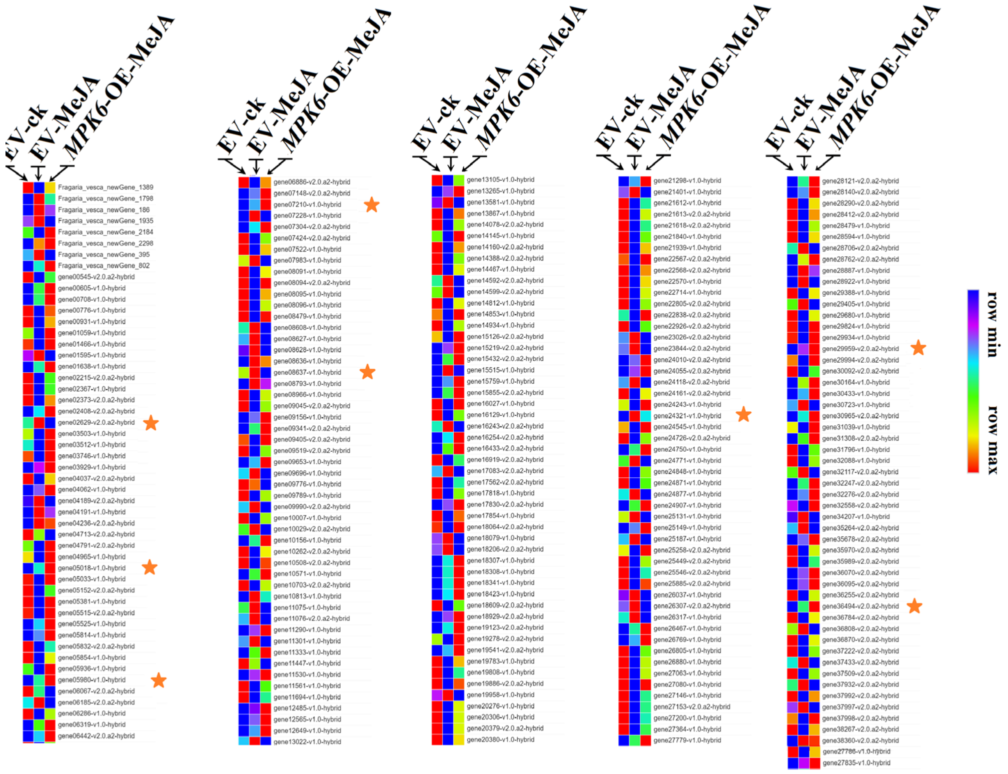

Supplementary Figure S2. heatmap showing the expression pattern of genes affected by both MeJA treatment and FaMPK6 over-expression. EV-CK, samples transformed with an empty vector with no MeJA treatment; EV-MeJA, samples transformed with an empty vector with MeJA treatment; MPK6-OE-MeJA; samples over-expressing FaMPK6 treated with MeJA.

GENE	#id	QRT-F	QRT-R
EXP2	gene21343-v1.0-hybrid	CGATCAAAGGCTCCAAGGGT	CGTCACTGGTTGTGACCTGA
EXP3	gene04435-v1.0-hybrid	TCCCTGACAAAATGGCTGCT	CATTTGCCACCGGGTTCTTG
CEL1	gene06191-v1.0-hybrid	TCCGAGCATAATCCAGTCGC	CTCGTTTTTGCCGGTGAAGG
PL	gene17555-v1.0-hybrid	TCTCATGCGCAACAGGGAAT	GTTGCGGCCGAATCCAATAC
PG	gene21638-v1.0-hybrid	ACTTCAACTGCGGAGGCTTT	GGGTTGCACGTTTGCACAAT
PE	gene12966-v1.0-hybrid	TGTGGACGTTGTGGTAGCTC	GCGCTTCAAGCTGTAATCCG
SUS1	gene12940-v1.0-hybrid	CATTGGCGGAAAAATCCCGT	CTCCTCGGCAACAAGTGAGT
SUS2	gene11077-v1.0-hybrid	GAAATCGTGGCGCTCCTTTC	GAGCCACAAATGGAGGGTGA
SPS3	gene31122-v1.0-hybrid	CCGAGGTGCCTTGGAGAAAT	ACCCCGCAGGGAAAGTTTAG
CS1	gene00583-v1.0-hybrid	AAATCCTCGACTTGCCCGAG	CTCCAGCAAGGGCAGTGTAA
ACO	gene23775-v1.0-hybrid	GCTCTCAGATCAGGGCCATC	GCAACCTATCGATCCTCGGG
MDH1	gene05605-v1.0-hybrid	ATGCTCTTTCACCCCGAAGG	AGCCAGCCCTCTCATACTCA
BAHD	gene33976-v1.0-hybrid	TGGAATTGAAGCGTGGGTGA	AGCGTCTTTGGAGATGCTAGT
COMT	gene01858-v2.0.a2-hybrid	TGGGATGTCATTTGTGCTAGGT	CCTTCAGTTTGGCAGCCATT
CSE	gene08444-v2.0.a2-hybrid	AAGCTTTTGTCTCGGACCCA	GGCTCTGTCTCAACAGAACAA
CCoAOMT	gene29535-v1.0-hybrid	CGCAGGACAGAACTGGCTAT	CAACAGGCTAGGGAAGCCAA
4CL	gene07129-v1.0-hybrid	TATAGCAGCCAAACCCGGTC	CGGCACTAGCTAGGGTTCTG
F5H	gene14889-v1.0-hybrid	TACAACAACCCGCGCCTATT	TTCGGGACCGGAAAAGTACG
CCR	gene29344-v2.0.a2-hybrid	TTGTCGCCTTCCAGTCTTCC	AGCTCCAGTTACACACACCG
PGIP1	gene00522-v1.0-hybrid	TGGTCAGATTCCGGTTGGTG	GCACAGACACCGGTTATGGA
CAD	gene24025-v2.0.a2-hybrid	TGGGTCACCATGTGACTGTG	TGGAAAGCCGGTACAGTGTC
PER4	gene15456-v1.0-hybrid	TGAGCCTCCACAATCCGAAG	GTCTTCCTCCTAGTATTGCATCG
bHLH74	gene02629-v2.0.a2-hybrid	GGCAGGCAAACAGACCAAAG	ATTTGTAGCCTGGCCCCTTC
MYB108	gene05018-v1.0-hybrid	GGAACTCTCTTGCTCGCTGT	GAACGTCTGGCCGGAGATAG
WRKY75	gene07210-v1.0-hybrid	ATGGGTTCTTGGGGCTGATG	ACGCATATCTCGGCTTCCTG
SalAT	gene08637-v1.0-hybrid	GTCGCGAGGCCATTTCAATC	ACCAGTATTTCGGTTTTACTTGAGG
TIFY5B	gene24321-v1.0-hybrid	GAGAAATGCAGCTCGGAGGA	AACATAAACCCGGCCATCGT
CYP450	gene29959-v1.0-hybrid	GCACTTCACCATGACCCTGA	ACAAGAGAGCAATTTCACAAGG
BGL12	gene36494-v2.0.a2-hybrid	TCAGGCTGAGCAGAAAGGTG	GAGATCTCATGCTGTGCGGA

Supplementary Table S1. Primers for quantitative real time (qRT)–PCR.
